# Transcriptomic Analysis of Persistent Infection with Foot-and-Mouth Disease Virus in Cattle Suggests Impairment of Apoptosis and Cell-Mediated Immunity in the Nasopharynx

**DOI:** 10.1371/journal.pone.0162750

**Published:** 2016-09-19

**Authors:** Michael Eschbaumer, Carolina Stenfeldt, George R. Smoliga, Juan M. Pacheco, Luis L. Rodriguez, Robert W. Li, James Zhu, Jonathan Arzt

**Affiliations:** 1 Plum Island Animal Disease Center (PIADC), Foreign Animal Disease Research Unit (FADRU), United States Department of Agriculture (USDA)—Agricultural Research Service (ARS), Greenport, NY, United States of America; 2 Oak Ridge Institute for Science and Education, PIADC Research Participation Program, Oak Ridge, TN, United States of America; 3 Animal Genomics and Improvement Laboratory (AGIL), USDA-ARS, Beltsville, MD, United States of America; University of Minnesota College of Veterinary Medicine, UNITED STATES

## Abstract

In order to investigate the mechanisms of persistent foot-and-mouth disease virus (FMDV) infection in cattle, transcriptome alterations associated with the FMDV carrier state were characterized using a bovine whole-transcriptome microarray. Eighteen cattle (8 vaccinated with a recombinant FMDV A vaccine, 10 non-vaccinated) were challenged with FMDV A_24_ Cruzeiro, and the gene expression profiles of nasopharyngeal tissues collected between 21 and 35 days after challenge were compared between 11 persistently infected carriers and 7 non-carriers. Carriers and non-carriers were further compared to 2 naïve animals that had been neither vaccinated nor challenged. At a controlled false-discovery rate of 10% and a minimum difference in expression of 50%, 648 genes were differentially expressed between FMDV carriers and non-carriers, and most (467) had higher expression in carriers. Among these, genes associated with cellular proliferation and the immune response–such as chemokines, cytokines and genes regulating T and B cells–were significantly overrepresented. Differential gene expression was significantly correlated between non-vaccinated and vaccinated animals (biological correlation +0.97), indicating a similar transcriptome profile across these groups. Genes related to prostaglandin E_2_ production and the induction of regulatory T cells were overexpressed in carriers. In contrast, tissues from non-carrier animals expressed higher levels of complement regulators and pro-apoptotic genes that could promote virus clearance. Based on these findings, we propose a working hypothesis for FMDV persistence in nasopharyngeal tissues of cattle, in which the virus may be maintained by an impairment of apoptosis and the local suppression of cell-mediated antiviral immunity by inducible regulatory T cells.

## Introduction

Foot-and-mouth disease is a highly contagious vesicular disease of cloven-hoofed animals [1] that is caused by foot-and-mouth disease virus (FMDV), a non-enveloped aphthovirus (family *Picornaviridae*). The single-stranded positive-sense RNA genome of FMDV is approximately 8.3 kb in length. It is polyadenylated and contains a single large open reading frame, which encodes a polyprotein that is subsequently cleaved into 4 structural and 8 non-structural proteins by viral proteases [2].

FMDV is a major concern for the international trade in animals and animal products, particularly because it can establish subclinical persistent infections in ruminants [3, 4]. Experimental studies with FMDV in cattle have reported 50% to 100% incidence of viral persistence, even in vaccinated animals that were fully protected against clinical disease [5–10].

Despite substantial research on FMDV persistence, little is known about how it is established and maintained [11]. The nasopharyngeal epithelium (NP) has been identified as a site of both primary [12–14] and persistent [8, 10, 15–18] FMDV infection in cattle; other studies have also reported the detection of persistent viral genome and antigen in regional lymph nodes of the nasopharynx [19–21].

Zhang and Alexandersen [22, 23] have suggested that the rate of viral clearance from a tissue, rather than the peak viral load during acute infection, is associated with persistence. Apoptotic processes, initiated from within infected cells or through effector molecules of natural killer cells and cytotoxic T lymphocytes, are essential for the removal of virus-infected cells [24]. An earlier study using the bovine transcriptome microarray suggested a potential role for the inhibition of apoptosis in persistent FMDV infection [25]. That study demonstrated relatively lower expression of pro-apoptotic genes in the NP compared to the lungs, which are permissive to FMDV replication during early infection [12] but do not become persistently infected [10].

Previous studies have investigated the expression of a limited set of candidate genes (TNF-α, IFN-α, β, and γ, IL-1α and β, IL-2, 4, 6, 10, 12, 15 and 18, CXCL10, CCL5, as well as TLR3 and TLR4) in the context of persistent FMDV infection, both on the macro- and the microanatomic scale [10, 17, 26, 27]. There is mounting evidence of suppression of antiviral host factors during persistent infection [10, 17], but no conclusive mechanism of FMDV persistence has been elucidated.

Research with other viruses (such as hepatitis B virus, hepatitis C virus and HIV) has revealed the common theme that during many chronic viral infections, antigen-specific T cells are initially activated and gain effector functions but progressively lose this functionality over time, a phenomenon called T-cell exhaustion [28]. Early defects in proliferation, IL-2 production and cytotoxicity are followed by the loss of TNF and IFN-γ production at late stages. With the increasing expression of inhibitory receptors like PD-1 and CTLA-4, exhausted cells become more responsive to inhibitory signals, resulting in further decreased effector function [29]. Apart from cell-surface inhibitory receptors, suppressive cytokines and regulatory T (T_reg_) cells are centrally involved in the pathogenesis of T-cell exhaustion [28, 30].

T_reg_ cells are a T cell subset involved in immune tolerance and homeostasis [31]. They comprise two main populations: natural and inducible T_reg_ cells [32]. Natural T_reg_ cells develop in the thymus and maintain tolerance to self-antigens. Outside of the thymus, other T cells can acquire regulatory function by antigenic stimulation in an appropriate cytokine milieu [31]. These inducible T_reg_ cells control immune homeostasis by suppressing effector T cells, particularly in the context of chronic infections [33]. However, many viruses exploit this mechanism to dampen immune responses allowing for viral persistence [30, 31, 34].

The present study compared the transcriptome profiles of NP tissues from persistently FMDV-infected carriers, non-carriers that had been previously infected but cleared the infection, and naïve controls. This work complements recent studies from our laboratory which describe the role of the systemic and regional [10] host response in FMDV persistence in the same cohorts of animals. This study provides the first thorough examination of the transcriptome of persistently FMDV-infected tissues, and proposes novel hypotheses about the mechanism of the FMDV carrier state divergence in cattle.

## Results

### Persistence status determination and tissue selection

The World Organisation for Animal Health (OIE) defines FMDV carriers as animals in which the virus persists for more than 28 days after initial infection [35]. In the present study, all probang samples from non-carriers were negative by virus isolation by 21 days post infection (dpi), whereas all animals that were positive by virus isolation on 21 dpi remained positive until 28 dpi and beyond [10]; there was no change in FMDV detection in probangs in any animal between 21 dpi and the end of the experiment. Thus, for the purposes of this study, FMDV persistence was defined by sustained detection of infectious virus in probang samples until at least 21 dpi, or until the day of necropsy, whichever was later.

Of 18 FMDV-infected cattle, 11 (61%) had detectable infectious virus in oropharyngeal fluid (OPF) throughout the study period and were classified as FMDV carriers. In each carrier animal, at least one of the four NP tissues tested was positive for both FMDV RNA and infectious virus; only tissues that fulfilled both criteria were used for the microarray analysis. All tissues from non-carriers were negative by virus isolation (Table 1).

**Table 1:** Tissue donor animals.

animal	FMDV status	vaccination	dpi	FMDV VI
106	FMDV carrier	non-vaccinated	21	positive
107	FMDV carrier	non-vaccinated	21	positive
030	FMDV carrier	non-vaccinated	35	positive
031	FMDV carrier	non-vaccinated	35	positive
034	FMDV carrier	non-vaccinated	35	positive
035	FMDV carrier	non-vaccinated	35	positive
110	FMDV carrier	non-vaccinated	35	positive
005	FMDV carrier	vaccinated (10X)	35	positive
013	FMDV carrier	vaccinated (10X)	35	positive
054	FMDV carrier	vaccinated (1X)	35	positive
056	FMDV carrier	vaccinated (1X)	35	positive
029	non-carrier	non-vaccinated	21	negative
032	non-carrier	non-vaccinated	35	negative
108	non-carrier	non-vaccinated	35	negative
015	non-carrier	vaccinated (10X)	35	negative
016	non-carrier	vaccinated (10X)	35	negative
055	non-carrier	vaccinated (1X)	35	negative
057	non-carrier	vaccinated (1X)	35	negative
047	naïve	non-vaccinated	—	—
048	naïve	non-vaccinated	—	—

Nasopharyngeal (NP) mucosa was collected postmortem from each animal. Animals are grouped by their FMDV carrier status. Based on the probang results, it was concluded that animals could be reliably categorized as either persistently infected FMDV carriers or non-carriers by 21 dpi [10]. (VI: virus isolation)

Substantial signal intensities for the FMDV probes on the microarray (58 of 43768 probes overall) were only found in tissue samples from persistently infected carriers, but not in samples from non-carriers.

### Differentially expressed genes

All 43768 probes on the array were included in the statistical analysis without pre-filtering. The estimated consensus intra-spot correlation for this experiment was 25.7%, and the observed differential expression between non-carriers and carriers was strongly correlated between vaccinated and non-vaccinated animals (Pearson’s r for fold changes +0.83, biological correlation +0.97; S1 Fig). On this basis, vaccination status was not analyzed further.

A biologically significant difference in expression of the corresponding gene was assumed for all probes with a p-value of <0.05, a q-value of <0.1 and an absolute (unsigned) log_2_ fold change (log_2_FC) >0.58, corresponding to a difference in signal intensity of at least 50% in either direction.

In the direct comparison between non-carriers and carriers, 867 probes (mapping to 648 unique genes; S1 Table) met the significance criteria; 656 (467) had higher intensities in carriers, and 211 (181) had higher intensities in non-carriers (Table 2).

**Table 2:** Total counts of probes with significantly different intensities.

comparison	q<0.1			q<0.1 and >±50% difference		
non-carriers vs. carriers	3092	1125↑	1967↓	867	211↑	656↓
non-carriers vs. naïve	1569	679↑	890↓	1276	556↑	720↓
carriers vs. naïve	4715	2440↑	2275↓	3637	1920↑	1717↓

For the direct comparison between non-carriers and carriers, ↑ indicates relatively higher intensity in non-carriers, and ↓ indicates relatively higher intensity in carriers (i.e., lower intensity in non-carriers). For the comparisons to the naïve control animals, the arrows indicate higher or lower intensity in the (previously) infected animals (non-carriers or carriers) than in the controls.

There was substantial overlap between the genes that were significantly up- or downregulated in non-carriers vs. controls and the genes that were significantly up- or downregulated in carriers vs. controls, because the expression of many genes was similar between both cohorts of FMDV-exposed animals (non-carriers and carriers) but different compared to the controls. The summary counts for differential expression between all non-carriers and the naïve controls (556 probes with higher intensity in non-carriers, 720 with lower) or between all carriers and the naïve controls (1920 with higher intensity in carriers, 1717 with lower) include all probes (Table 2). Among the 867 probes with significantly different intensities between non-carriers and carriers, only 11 were significantly different between non-carriers and naïve animals (10/1), whereas 329 were significantly different between carriers and naïve animals (303/26) (S2 and S3 Tables).

It is noteworthy that while there were 11 FMDV carrier animals and 7 non-carriers, the study included only 2 naïve controls; thus, the direct comparison between carriers and non-carriers has more statistical power than the comparisons to the naïve controls, and the analysis of the data is focused on the former.

**Fig 1:**
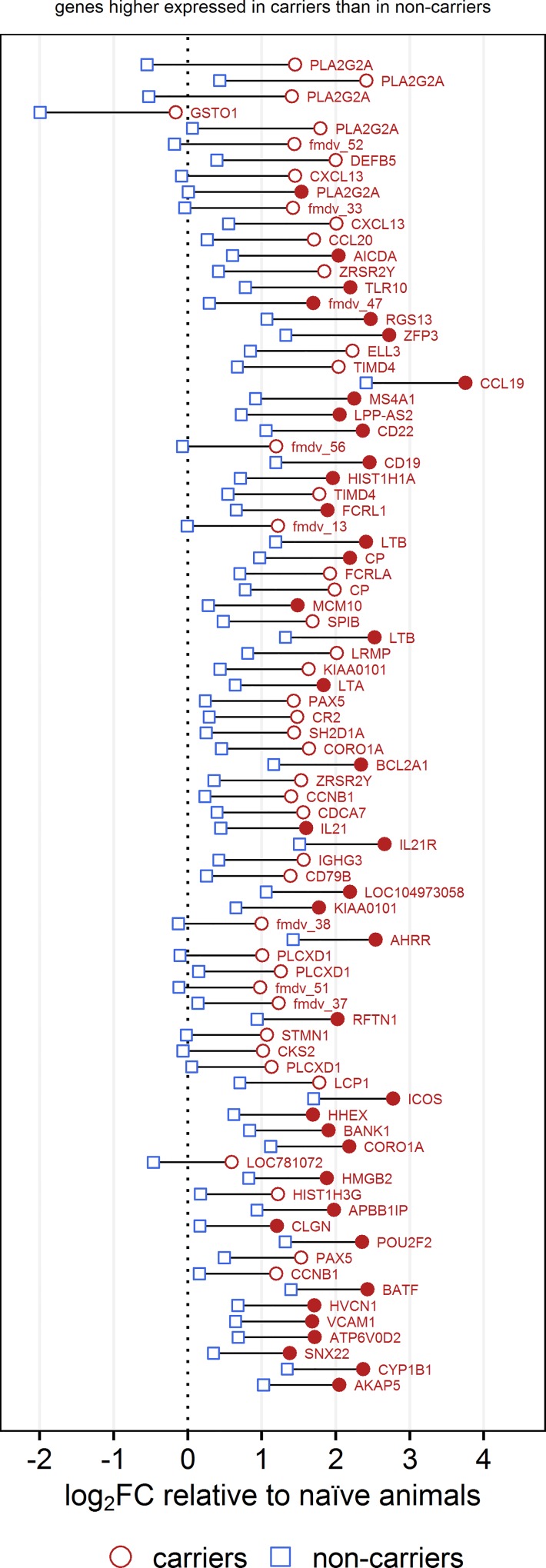
Differential gene expression between non-carriers, carriers and naïve controls: Genes higher expressed in carriers. The 100 probes with the largest difference in expression between non-carriers and carriers (out of a total of 867 with q<0.1) are shown ordered by decreasing difference. Genes that were expressed higher in carriers are shown in Fig 1, and genes that were higher expressed in non-carriers are shown in Fig 2. For each probe, the fold change relative to the naïve controls is shown on the x-axis with the vertical dashed line representing no change compared to the naïve animals. Fold changes in signal intensity between non-carriers and naïve controls are marked with blue squares, and the fold changes between carriers and naïve animals are marked with red circles. Filled blue or red symbols indicate a significant difference in intensity (q<0.1) compared to the naïve animals. The horizontal distance between each square and circle represents the difference in signal intensity between non-carriers and carriers, and the color of the label indicates the group in which the signal intensity was higher (blue for non-carriers, red for carriers). The difference between non-carriers and carriers is significant (q<0.1) for all probes shown, independent of whether the difference between each infected group and the controls is significant.

**Fig 2:**
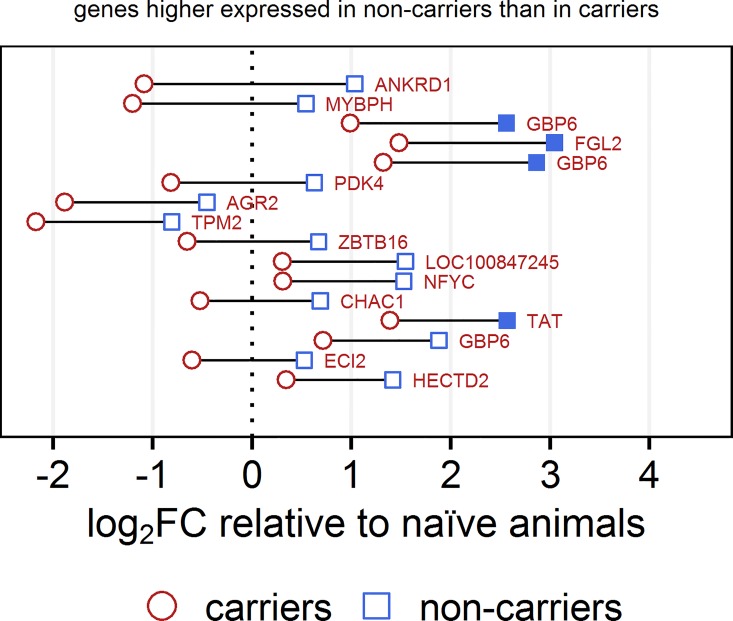
Differential gene expression between non-carriers, carriers and naïve controls: Genes higher expressed in non-carriers. The 100 probes with the largest difference in expression between non-carriers and carriers (out of a total of 867 with q<0.1) are shown ordered by decreasing difference. Genes that were expressed higher in carriers are shown in Fig 1, and genes that were higher expressed in non-carriers are shown in Fig 2. For each probe, the fold change relative to the naïve controls is shown on the x-axis with the vertical dashed line representing no change compared to the naïve animals. Fold changes in signal intensity between non-carriers and naïve controls are marked with blue squares, and the fold changes between carriers and naïve animals are marked with red circles. Filled blue or red symbols indicate a significant difference in intensity (q<0.1) compared to the naïve animals. The horizontal distance between each square and circle represents the difference in signal intensity between non-carriers and carriers, and the color of the label indicates the group in which the signal intensity was higher (blue for non-carriers, red for carriers). The difference between non-carriers and carriers is significant (q<0.1) for all probes shown, independent of whether the difference between each infected group and the controls is significant.

For the 100 probes with the largest difference between non-carriers and carriers, the magnitude and directionality of the differential expression are presented in detail (Figs 1 and 2). The set of 656 probes that had significantly higher signal intensities in carriers included 34 FMDV genome-specific probes (indicated by the “fmdv” prefix in Fig 1 and the supplemental tables).

### Biological pathway analysis

All genes that were differentially expressed between FMDV carriers and non-carriers were subjected to a “hypothesis-free” enrichment analysis using Gene Ontology (GO) terms and the Kyoto Encyclopedia of Genes and Genomes (KEGG). Among the genes that were more highly expressed in non-carriers than in carriers, 13 GO terms and 2 KEGG pathways were significantly overrepresented. These terms were generally related to cellular metabolism (organic acid metabolic process, negative regulation of metabolic process, regulation of fatty acid oxidation, transaminase activity, 2-oxocarboxylic acid metabolism and biosynthesis of amino acids) or differentiation (tissue development, goblet cell differentiation, and organ morphogenesis, transcription factor activity), but the enrichment analysis provided no further leads.

Conversely, among the genes that were expressed more highly in FMDV carriers compared to non-carriers, 14 GO terms for biological processes (BP), cellular components (CC) and molecular functions (MF), as well as 15 KEGG pathways were significantly overrepresented (Table 3). Fifteen of the 29 identified terms/pathways are immune related.

**Table 3:** Overrepresented functional terms among genes that are expressed more highly in persistently infected FMDV carriers (compared to non-carriers).

term ID	type	Description	p-value
GO:0000280	BP	nuclear division	1.61E-23
GO:0034502	BP	protein localization to chromosome	9.00E-04
GO:0060326	BP	cell chemotaxis	2.62E-03
GO:0048247	BP	lymphocyte chemotaxis	6.46E-03
GO:0001816	BP	cytokine production	1.22E-02
GO:0051707	BP	response to other organism	3.16E-02
GO:0070098	BP	chemokine-mediated signaling pathway	3.96E-02
GO:0005694	CC	Chromosome	2.34E-28
GO:0009897	CC	external side of plasma membrane	2.16E-09
GO:0042101	CC	T-cell receptor complex	5.75E-05
GO:0005515	MF	protein binding	7.09E-07
GO:0003677	MF	DNA binding	4.08E-05
GO:0048020	MF	CCR chemokine receptor binding	2.25E-03
GO:0003777	MF	microtubule motor activity	1.49E-02
KEGG:04110	PW	cell cycle	2.23E-09
KEGG:05322	PW	systemic lupus erythematosus	6.54E-07
KEGG:05166	PW	HTLV-I infection	7.22E-06
KEGG:04060	PW	cytokine-cytokine receptor interaction	1.64E-05
KEGG:03410	PW	base excision repair	2.44E-05
KEGG:05340	PW	primary immunodeficiency	1.87E-04
KEGG:04064	PW	NF-κB signaling pathway	2.40E-04
KEGG:04662	PW	B-cell-receptor signaling pathway	1.05E-03
KEGG:04660	PW	T-cell-receptor signaling pathway	3.72E-03
KEGG:03030	PW	DNA replication	7.13E-03
KEGG:04062	PW	chemokine signaling pathway	2.03E-02
KEGG:04640	PW	hematopoietic cell lineage	2.29E-02
KEGG:04514	PW	cell adhesion molecules	3.42E-02
KEGG:05202	PW	transcriptional misregulation in cancer	4.39E-02
KEGG:04672	PW	intestinal immune network for IgA production	4.58E-02

BP: biological process, CC: cellular component, MF: molecular function, PW: pathway. Overrepresented terms identified by g:Profiler are ordered by increasing p-value within each category.

**Fig 3:**
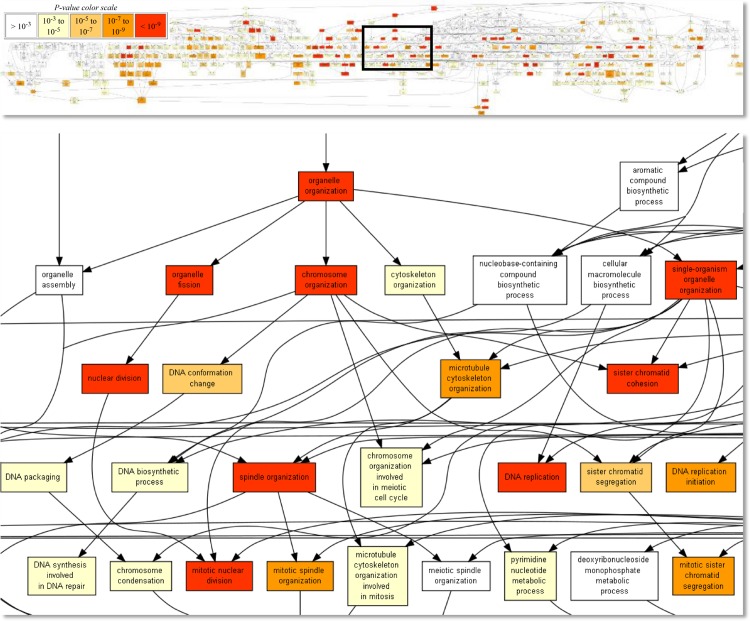
Overrepresented Gene Ontology terms among genes that were higher expressed in carriers: Terms related to cellular proliferation. Taken from the output of the GOrilla web tool. Fields are colored by p-value, from >10^-3^ (white) to <10^-9^ (red).

**Fig 4:**
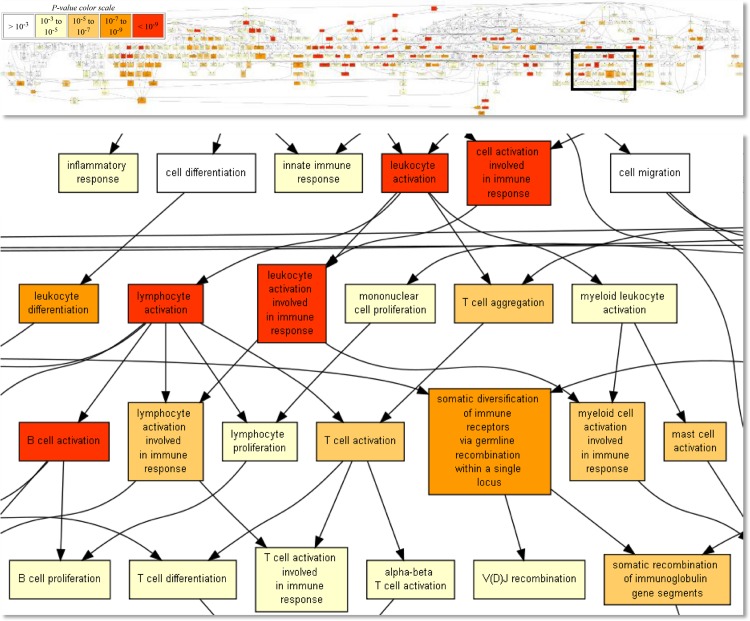
Overrepresented Gene Ontology terms among genes that were higher expressed in carriers: Terms related to the adaptive immune response. Taken from the output of the GOrilla web tool. Fields are colored by p-value, from >10^-3^ (white) to <10^-9^ (red).

The overrepresented functional terms for carriers were roughly grouped into two cohesive clusters–cellular proliferation (Fig 3) and immune responses (Fig 4)–suggesting an increase of related biological activity in persistently FMDV-infected tissues.

Of note, genes in both the B-cell-receptor-signaling pathway (DAPP1, CD22, PIK3CD, BTK, CR2, and CD79B) and the T-cell-receptor signaling pathway (ITK, ZAP70, PDCD1, CD247, CTLA4, CD3E, PIK3CD, LAT, ICOS, and RASGRP1) were overrepresented. Together with findings from an earlier study with the bovine microarray [25], these genes served as starting points for a “candidate gene” approach to the data analysis.

### Death receptor signaling

Apoptosis induced by death receptor signaling is one of the immune mechanisms that eliminate infected cells [24]. Based on our previous finding that tissues that are not susceptible to persistent FMDV infection have higher expression of death receptor signaling genes [25], we examined if the expression of death receptors and their ligands was also higher in the tissues from non-carriers compared to carriers. Based on signal intensity, death receptor 6 (DR6), a pro-apoptotic member of the TNF receptor superfamily [36] (encoded by TNFRSF21) was the highest expressed death receptor in the NP tissues in this study. DR6 was significantly overexpressed in NP tissues from non-carriers compared to carriers (Table 4), as were amyloid precursor protein β (APP) and beta-secretases (BACE1 and BACE2), which cleave a DR6 ligand peptide from APP [37].

**Table 4:** Death receptors and their ligands.

gene	log_2_ FC (NC/CR)	p	q	avg. signal
tumor necrosis factor α (TNF)	-0.06	0.64	0.86	177
tumor necrosis factor α, natural antisense transcript (TNF-AS1)	-0.66	**<0.01**	**0.07**	89
TNF receptor superfamily, member 1A (TNFRSF1A), TNFR1	+0.24	0.05	0.27	1406
TNF receptor superfamily, member 1B (TNFRSF1B), TNFR2	-0.33	0.06	0.29	1940
TNF receptor-associated factor 1 (TRAF1)	-0.37	**<0.01**	**0.06**	1168
TNF receptor-associated factor 2 (TRAF2)	-0.05	0.52	0.79	2218
baculoviral IAP repeat-containing 3, cIAP2 (BIRC3)	-0.81	**<0.01**	**0.05**	538
BCL2-related protein A1 (BCL2A1)	-1.18	**0.01**	**0.09**	825
amyloid beta (A4) precursor protein (APP)	+0.55	**0.01**	0.12	2978
beta-site APP-cleaving enzyme 1, beta-secretase (BACE1)	+0.34	**0.01**	**0.09**	121
beta-site APP-cleaving enzyme 2, theta-secretase (BACE2)	+0.64	**0.03**	**0.19**	1239
TNF receptor superfamily, member 21, DR6 (TNFRSF21)	+0.59	**<0.01**	**0.03**	4991

Negative log_2_ FC values indicate higher expression in tissues from persistently infected FMDV carriers (CR) compared to non-carriers (NC). All p- and q-values that met the established significance criteria are marked in bold. (p: unadjusted p-value at gene level, q: transcriptome-wide p-value after Benjamini-Hochberg adjustment, avg. signal: average intensity of this probe across all arrays)

Tumor necrosis factor α (TNF-α) binding to TNF receptors potentially initiates apoptosis [24]. TNF-AS1, a probe designed based on TNF antisense transcripts (accession numbers FE004591, FE004592, DN541181 and DN543283; see http://www.ncbi.nlm.nih.gov/dbEST and S2 Fig), had significantly higher intensity in carriers than in non-carriers. The function of these TNF antisense transcripts is unknown, but antisense transcripts that are reverse complementary to the translation initiation site can interfere with gene expression [38]. Thus, the overexpression of this factor further supports a relatively anti-apoptotic state in FMDV carriers.

Among known TNFα receptors, TNFRSF1A (TNFα receptor 1), which contains a death domain [39], was more highly expressed in non-carriers. Conversely, the TNFα receptor 2 (TNFRSF1B), lacking the death domain [40], was more highly expressed in carriers, as were the TNF receptor-associated protein TRAF1 and its interaction partner BIRC3 (Table 4). BIRC3 is an inhibitor of apoptosis protein that interferes with caspase activation [39]. Bfl-1/A1 (BCL2A1), a transcriptional target of nuclear factor κB (NF-κB) that suppresses caspase activation and apoptosis in response to death-inducing stimuli like TNFα [41], was also more highly expressed in carriers than in non-carriers.

Pro-apoptotic genes that were expressed significantly higher in non-carriers than in carriers included ankyrin repeat domain 1 (ANKRD1), glutathione-specific γ-glutamylcyclotransferase (CHAC1) [42], and the tumor suppressor gene OSR1 (Table 5). The expression of OSR1 was significantly reduced in carriers compared to naïve controls (S3 Table); its knockdown *in vitro* inhibits apoptosis [43]. In the direct comparison between FMDV carriers and non-carriers, ANKRD1 was the most strongly overexpressed gene in the non-carriers. ANKRD1 encodes a pleiotropic protein of a conserved family of ankyrin-repeat proteins that interferes with transforming growth factor (TGF) β signaling [44] and promotes apoptosis [45]. Overall, these results suggest that differences in the expression of genes involved in death receptor signaling and apoptosis may play an important role in the FMDV carrier/non-carrier divergence.

**Table 5:** Other proapoptotic genes.

Gene	log_2_ FC (NC/CR)	p	q	avg. signal
ankyrin repeat domain 1 (ANKRD1)	+2.12	**<0.01**	**0.05**	104
ChaC glutathione-specific γ-glutamylcyclotransferase (CHAC1)	+1.21	**<0.01**	**0.03**	224
odd-skipped related 1 (OSR1)	+0.88	**<0.01**	**0.04**	1366

Negative log_2_ FC values indicate higher expression in tissues from persistently infected FMDV carriers (CR) compared to non-carriers (NC). All p- and q-values that met the established significance criteria are marked in bold. (p: unadjusted p-value at gene level, q: transcriptome-wide p-value after Benjamini-Hochberg adjustment, avg. signal: average intensity of this probe across all arrays)

### Cellular immunity

Cell-mediated immunity is an important mechanism for the clearance of infected cells and a highly regulated process. Among the overrepresented T-cell-receptor signaling genes, PD-1 (encoded by PDCD1) and CTLA-4 are important inhibitory receptors that are involved in T-cell exhaustion [46], which is commonly associated with persistent viral infections [47]. Functional effector T cells can transiently express inhibitory receptors during activation and PD-1 is constitutively expressed by follicular T-helper cells [48]. High expression of multiple inhibitory receptors, however, is a key feature of the exhaustion of CD4 and CD8 T cells [28]. PD-1 and other cell surface inhibitory receptors as well as transcription factors that co-regulate T-cell exhaustion (CTLA-4, LAG-3, BTLA, and Tim-3, BATF, NFAT1 and eomesodermin) [47, 49] were all significantly overexpressed in NP tissues from persistently FMDV-infected carriers (Table 6).

**Table 6:** Inhibitory receptors and transcription factors associated with T-cell exhaustion.

Gene	log_2_ FC (NC/CR)	p	q	avg. signal
programmed cell death 1, PD-1 (PDCD1)	-0.67	**<0.01**	**0.06**	577
cytotoxic T-lymphocyte-associated 4, CD152 (CTLA4)	-0.86	**<0.01**	**0.04**	80
lymphocyte-activation gene 3, LAG-3 (LAG3)	-0.68	**<0.01**	**0.06**	76
B and T lymphocyte associated, CD272 (BTLA)	-0.71	**<0.01**	**0.03**	40
hepatitis A virus cellular receptor 2, Tim-3 (HAVCR2)	-0.21	**0.05**	0.25	48
CD160 antigen (CD160)	+0.11	0.22	0.54	48
natural killer cell receptor 2B4 (CD244)	-0.03	0.72	0.90	35
T cell immunoreceptor with Ig and ITIM domains (TIGIT)	-0.44	0.11	0.38	405
basic leucine zipper transcription factor, ATF-like (BATF)	-0.42	**<0.01**	**0.05**	31
nuclear factor of activated T-cells, NFAT1 (NFATC2)	-0.37	**0.02**	0.17	187
eomesodermin homolog (EOMES)	-0.56	**<0.01**	**0.05**	46
T-cell-specific T-box transcription factor, T-Bet (TBX21)	-0.37	0.06	0.28	105

Negative log_2_ FC values indicate higher expression in tissues from persistently infected FMDV carriers (CR) compared to non-carriers (NC). All p- and q-values that met the established significance criteria are marked in bold. (p: unadjusted p-value at gene level, q: transcriptome-wide p-value after Benjamini-Hochberg adjustment, avg. signal: average intensity of this probe across all arrays)

T-cell exhaustion is caused by chronic antigenic stimulation of T-cells in an immunosuppressive cytokine milieu [49], and T_reg_ cells can contribute to this process through the production of IL-10 and the induction of tolerogenic DCs [50]. The genes for TGFβ and IL-10 as well as many cell surface and intracellular molecules associated with type 1 inducible T_reg_ (Tr1) cells (LAG-3, TNFRSF18/GITR, TNFRSF9/CD137, ICOS/CD278, ITGB2/CD18 and the transcription factors MAF/c-Maf, ZBTB32/ROG, EGR2, STAT3 and STAT5A [51]) were significantly overexpressed in NP tissues from carriers (Table 7). Tr1 cells are a subset of T cells that have strong immunosuppressive properties. They suppress effector T cells via IL-10- and TGFβ-dependent mechanisms, but do not express Forkhead box 3 (FOXP3), the signature transcription factor of natural T_reg_ cells [52, 53]. In contrast to natural T_reg_ cells, which originate in the thymus, Tr1 cells are induced in the periphery by exposure to their specific antigen in the presence of inhibitory cytokines [54].

**Table 7:** Stimulating cytokines, functional markers and recruiting chemokines of regulatory T cells.

Gene	log_2_ FC (NC/CR)	p	q	avg. signal
interleukin (IL) 10 (IL10)	-0.34	**0.02**	0.18	43
transforming growth factor β1 (TGFB1)	-0.39	**<0.01**	**0.05**	85
lymphocyte-activation gene 3 (LAG3)	-0.68	**<0.01**	**0.06**	76
TNF receptor superfamily, member 18, GITR (TNFRSF18)	-0.91	**<0.01**	**0.07**	488
TNF receptor superfamily, member 4, OX40 (TNFRSF4)	-0.30	0.10	0.37	701
TNF receptor superfamily, member 9, CD137 (TNFRSF9)	-0.85	**<0.01**	**0.03**	55
inducible T-cell co-stimulator, CD278 (ICOS)	-1.08	**0.01**	**0.10**	563
integrin β2, CD18 (ITGB2)	-0.48	**0.02**	0.17	66
IL-12/IL-35 α-chain (IL12A)	-0.36	**0.01**	0.12	188
Epstein-Barr virus induced gene 3, IL-27/IL-35 β-chain (EBI3)	-0.57	**<0.01**	**0.04**	56
IL-27 α-chain (IL27)	+0.18	**0.03**	0.21	30
IL-2 receptor, α-chain, CD25 (IL2RA)	-0.55	**<0.01**	**0.05**	47
forkhead box P3 (FOXP3)	-0.02	0.80	0.93	23
fibrinogen-like 2 (FGL2)	+1.57	**<0.01**	**0.03**	79
V-maf oncogene homolog, c-Maf (MAF)	-0.44	**0.01**	0.12	193
zinc finger and BTB domain containing 32, ROG (ZBTB32)	-0.81	**0.01**	0.13	150
early growth response 2 (EGR2)	-0.69	**0.01**	0.13	163
aryl hydrocarbon receptor (AHR)	-0.21	0.09	0.35	119
signal transducer and activator of transcription 3 (STAT3)	+0.61	**<0.01**	**0.03**	1449
signal transducer and activator of transcription 4 (STAT4)	-0.56	**<0.01**	**0.05**	101
signal transducer and activator of transcription 5A (STAT5A)	-0.25	**0.02**	0.14	633
chemokine (C-C motif) ligand 17 (CCL17)	-0.07	0.29	0.61	29
chemokine (C-C motif) ligand 22 (CCL22)	-0.48	**<0.01**	**0.05**	79
interleukin 16 (IL16)	-0.93	**0.01**	0.13	1336
interleukin 21 (IL21)	-1.15	**<0.01**	0.03	78

Negative log_2_ FC values indicate higher expression in tissues from persistently infected FMDV carriers (CR) compared to non-carriers (NC). All p- and q-values that met the established significance criteria are marked in bold. (p: unadjusted p-value at gene level, q: transcriptome-wide p-value after Benjamini-Hochberg adjustment, avg. signal: average intensity of this probe across all arrays)

Several of the overrepresented functional terms in carriers (Table 3) involve cytokine and chemokine signaling. Specifically, IL-16 and the macrophage-derived chemokine CCL22 were significantly higher expressed in persistently FMDV-infected NP tissues (Table 7). IL-16 and CCL22 preferentially attract T_reg_ cells, and CCL22 also attracts T_H_2-polarized T lymphocytes [55, 56]. IL-21, an important GC cytokine produced by follicular T helper cells [57], was significantly higher expressed in NP tissues from carriers. IL-21 has a suppressive effect on FOXP3^+^ natural T_reg_ cells, but induces FOXP3^-^ Tr1 cells [48, 52].

Both components of the IL-35 heterodimer (IL12A and EBI2) were significantly higher expressed in carriers (Table 7). IL-35 is primarily expressed by T_reg_ cells and is directly involved in their suppressive activity [58]. T_reg_ cell-derived IL-35 promotes T-cell exhaustion [59], and binding of IL-35 can induce the conversion of effector T cells to iTr35 T_reg_ cells, which suppress effector T cells in an IL-10-, TGFβ- and contact-independent manner [58].

**Fig 5:**
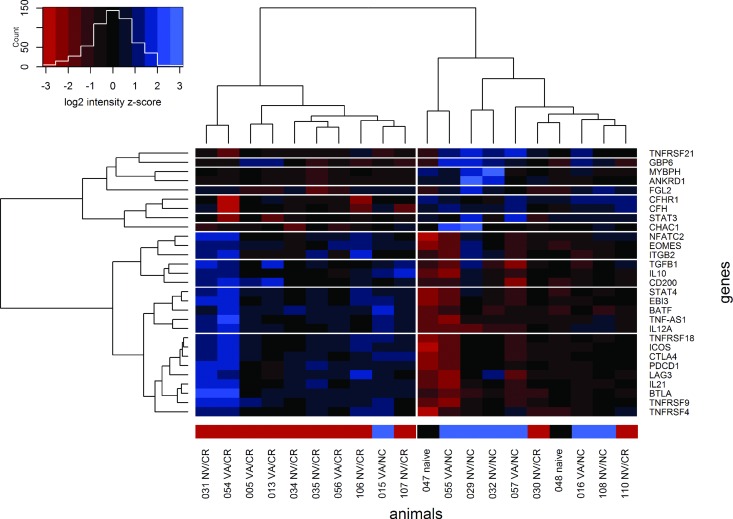
Heat map of signal intensities for selected probes. The ordering of the probes (rows) and animals (columns) is based on an unsupervised cluster analysis. The associated dendrograms are shown to the left and above the heat map. The colors in the heat map represent centered and scaled intensity values. Cells with negative z-scores (intensities lower than the overall mean for any given probe) are shaded red and cells with positive z-scores (higher intensities) are shaded blue. The colored sidebar above the heat map indicates the persistence status of each animal (carriers are shown in red, non-carriers in blue and controls in black).

Overall, these results indicate that inducible T_reg_ cells (Tr1 and iTr35) may be the cause of T-cell exhaustion and the impairment of cell-mediated immunity in persistently FMDV-infected carriers. The expression of many genes associated with Tr1 cells and T-cell exhaustion (PDCD1, CTLA4, LAG3 TNFRSF18/GITR and ICOS) was closely correlated in the FMDV carrier animals. Based on the signal intensities of selected probes (genes) related to apoptosis, T-cell exhaustion, T_reg_ cells, prostaglandin synthesis and the alternative complement pathway, 9 out of 11 carriers and 6 out of 7 non-carriers were correctly grouped by an unsupervised clustering algorithm (Fig 5).

### Humoral immunity

Many of the genes that were overexpressed in carriers were related to a strong T_H_2 or humoral immune response. In particular, there was substantial evidence suggesting an activation of B-cell follicles and germinal centers (GC) in the mucosa-associated lymphoid tissue (MALT) based on the observed high expression of B-cell trophic factors (Table 8). GCs are specialized structures that form in the follicular areas of secondary lymphoid organs. Two homeostatic chemokine systems with important roles in GC development and function [60, 61] were overexpressed in carriers (LTA/LTB, CXCR5/CXCL13 and CCR7/CCL19/CCL21). CCL19 was the gene that was most strongly overexpressed in carriers compared to naïve controls (S3 Table).

**Table 8:** B-cell-related genes.

Gene	log_2_ FC (NC/CR)	p	q	avg. signal
interleukin 21 (IL21)	-1.15	**<0.01**	**0.03**	78
interleukin 21 receptor (IL21R)	-1.15	**0.01**	**0.10**	1201
CD40 ligand, CD154 (CD40LG)	-0.52	**<0.011**	**0.048**	41
lymphotoxin alpha, tumor necrosis factor beta (LTA)	-1.20	**<0.01**	**0.05**	192
lymphotoxin beta (TNF superfamily, member 3) (LTB)	-1.23	**<0.01**	**0.05**	1518
chemokine (C-X-C motif) receptor 5 (CXCR5)	-1.42	**0.01**	0.11	1503
chemokine (C-X-C motif) ligand 13 (CXCL13)	-1.53	**<0.01**	**0.04**	151
chemokine (C-C motif) receptor 7 (CCR7)	-1.02	**<0.01**	**0.06**	177
chemokine (C-C motif) ligand 19 (CCL19)	-0.67	**<0.01**	**0.06**	60
chemokine (C-C motif) ligand 21 (CCL21)	-0.19	0.07	0.30	603
CD19 molecule (CD19)	-0.98	**<0.01**	**0.04**	94
membrane-spanning 4-domains, A1 (MS4A1), CD20	-1.34	**<0.01**	**0.08**	3209
complement component receptor 2 (CR2), CD21	-1.19	**0.01**	**0.09**	374
CD79b molecule (CD79B)	-1.14	**<0.011**	**0.05**	135
raftlin, lipid raft linker 1 (RFTN1)	-1.09	**<0.01**	**0.03**	1312
activation-induced cytidine deaminase (AICDA)	-1.44	**<0.01**	**0.05**	165
nuclear GTPase, germinal center associated (NUGGC)	-0.93	**<0.01**	**0.05**	59
regulator of G-protein signaling 13 (RGS13)	-1.40	**<0.01**	**0.05**	177

Negative log_2_ FC values indicate higher expression in tissues from persistently infected FMDV carriers (CR) compared to non-carriers (NC). All p- and q-values that met the established significance criteria are marked in bold. (p: unadjusted p-value at gene level, q: transcriptome-wide p-value after Benjamini-Hochberg adjustment, avg. signal: average intensity of this probe across all arrays)

Other GC-associated genes that were overexpressed in carriers include IL-21 (produced by follicular helper T cells) [57], the regulator of G-protein signaling 13 (RGS13) [62] and GC-associated nuclear GTPase (NUGGC) [63]. The B cell antigens CD19, CD20 (MS4A1), CD21 (CR2), the B-cell-receptor-associated proteins CD79b [64], activation-induced cytidine deaminase (AICDA) [65] and lipid raft linker 1 (RFTN1) [64] as well as the IL-21 receptor (IL21R) [57] were also highly overexpressed in carriers (Table 8).

### Innate immunity

The innate immune system is a critical component of host immunity, with great influence on the development of the adaptive immune response. Among the genes that were differentially expressed between carriers and non-carriers, group IIA phospholipase A2 (PLA2G2A) and complement factor H (CFH) were the most prominent innate immune effector genes.

Phospholipase A2 is an antimicrobial peptide [66] and a key enzyme in the production of prostaglandins and other eicosanoids [67]. It was highly overexpressed in carriers, and five of the nine probes with the largest difference in signal intensity between carriers and non-carriers mapped to this gene. PLA2G2A and glutathione S-transferase (GSTO1) were the most strongly overexpressed genes in carriers compared to non-carriers (Fig 1).

In addition to PLA2G2A, genes for other enzymes in the prostaglandin E_2_ (PGE_2_) and leukotriene (LTE) synthesis pathways were more highly expressed in carriers (Table 9). Persistently FMDV-infected tissues expressed significantly more cytosolic prostaglandin E synthase (PTGES3) and multidrug resistance protein 4, the main efflux transporter of PGE_2_ [68] than tissues from non-carriers. In addition, two key enzymes in the LTE pathway [69], arachidonate 5-lipoxygenase (ALOX5) and GSTO1, as well as the LTE receptor CYSLTR1 [70], were more highly expressed in carriers than in non-carriers, suggesting that persistently FMDV-infected tissues produce higher levels of the inflammatory mediators PGE_2_ and LTE.

**Table 9:** Eicosanoid synthesis and T_H_2 polarization.

Gene	log_2_ FC (NC/CR)	p	q	avg. signal
phospholipase A2, group IIA (PLA2G2A)	-2.00	**<0.01**	**0.03**	732
prostaglandin G/H synthase and cyclooxygenase (PTGS2)	-0.33	0.09	0.35	54
prostaglandin E synthase 3 (cytosolic) (PTGES3)	-0.47	**<0.01**	**0.06**	139
multi-drug resistance protein 4 (ABCC4)	-0.40	**0.04**	0.24	50
arachidonate 5-lipoxygenase (ALOX5)	-0.52	**<0.01**	**0.03**	178
glutathione S-transferase omega 1 (GSTO1)	-1.83	**<0.01**	**0.03**	1277
cysteinyl leukotriene receptor 1 (CYSLTR1)	-0.43	**<0.01**	**0.03**	41
interferon, gamma (IFNG)	+0.13	0.46	0.75	35
interleukin 4 (IL4)	-0.17	**<0.01**	**0.08**	22

Negative log_2_ FC values indicate higher expression in tissues from persistently infected FMDV carriers (CR) compared to non-carriers (NC). All p- and q-values that met the established significance criteria are marked in bold. (p: unadjusted p-value at gene level, q: transcriptome-wide p-value after Benjamini-Hochberg adjustment, avg. signal: average intensity of this probe across all arrays)

Complement factor H (CFH), CFH-related proteins and CD46 are regulators of the alternative complement activation pathway that protect host cells from damage caused by overactivation of the complement system [71]. These genes were significantly higher expressed in the non-carriers than in the carriers (Table 10), and this difference was mostly caused by a significant downregulation in carriers compared to the controls (S3 Table). A member of the immunomodulating guanylate binding protein family, GBP6, was highly overexpressed in non-carriers compared to carriers and controls. Three of the 15 probes with the largest difference in signal intensity between non-carriers and carriers mapped to this gene (Fig 2).

**Table 10:** The complement system and other innate immunity genes.

Gene	log_2_ FC (NC/CR)	p	q	avg. signal
complement component 3 (C3)	+0.57	0.06	0.30	12589
complement factor H (CFH)	+0.65	**<0.01**	**0.02**	775
complement factor H-related 1 (CFHR1)	+0.75	**<0.01**	**0.04**	634
decay accelerating factor for complement (CD55)	+0.37	**0.02**	0.16	633
complement factor I (CFI)	+0.45	0.05	0.27	720
complement factor D (CFD)	+0.35	**0.04**	0.24	1771
complement regulatory protein (CD46)	+0.35	**<0.01**	**0.05**	2164
CD200 antigen, OX2 (CD200)	-0.73	**<0.01**	**0.02**	412
CD200 receptor 1 (CD200R1)	-0.22	**0.04**	0.22	54
toll-like receptor 10 (TLR10)	-1.42	**<0.01**	**0.05**	293
guanylate binding protein family, member 6 (GBP6)	+1.54	**<0.01**	**0.03**	74

Negative log_2_ FC values indicate higher expression in tissues from persistently infected FMDV carriers (CR) compared to non-carriers (NC). All p- and q-values that met the established significance criteria are marked in bold. (p: unadjusted p-value at gene level, q: transcriptome-wide p-value after Benjamini-Hochberg adjustment, avg. signal: average intensity of this probe across all arrays)

Toll-like receptors are another key component of the innate immune system. TLR10 was significantly more highly expressed in carriers, both when compared to non-carriers and to naïve controls. TLR10 is an anti-inflammatory pattern recognition receptor that is also expressed on T_reg_ cells [72, 73]. Similarly, CD200 (OX2) and its receptor CD200R, both also overexpressed in carriers, have broad inhibitory effects on innate and adaptive immunity. CD200R activation mediates the polarization of effector T cells into Tr1 cells producing IL-10 and TGFβ, modulates the cytokine environment from T_H_1 to T_H_2, and facilitates the synthesis of anti-inflammatory mediators [74]. Many pathogens exploit the CD200/CD200R signaling pathway, e.g. to restrict viral-induced inflammation during respiratory influenza infection or to interfere with the control of coronavirus infection [75].

Taken together, the observed differences in the expression of genes of the innate, cellular and humoral response suggest an impairment of T_H_1 immunity in the nasopharynx of persistently infected FMDV carrier animals that is driven by the immunosuppressive effects of inducible Tr1 T_reg_ cells.

## Discussion

This study compared gene expression profiles in nasopharyngeal (NP) tissues of cattle persistently infected with FMDV (carriers) and cattle that had been infected with FMDV but cleared the infection (non-carriers). This is the first time that tissues from FMDV carriers and non-carriers were analyzed with a bovine whole-transcriptome microarray, and we identified several sets of differentially expressed genes that could explain the mechanisms underlying the persistence or clearance of FMDV infection in these animals. Many of the genes that were more highly expressed in carriers suggest a broad activation of immune cells in persistently FMDV-infected tissues. Apoptosis plays a vital role in immune homeostasis [76], and the observed regulation of pro- and anti-apoptotic genes could be a consequence of immune system activation with or without impact on FMDV persistence. Despite the novelty of these findings, this work should be considered to be an effort towards generating hypotheses based upon a single modality of investigation, the bovine whole-transcriptome microarray. Any hypotheses proposed herein must be subjected to additional validation, including quantification of mRNA by qRT-PCR or next-generation sequencing as well as characterization of the associated proteins by flow cytometry or immunomicroscopy.

The findings of the present study support the previously proposed concept of a connection between the ability to eliminate infected cells via death receptor signaling and the susceptibility to persistent FMDV infection [25]. To clear a virus infection, infected cells must be safely eliminated before infectious virus progeny is released [24]. This is achieved via immune mechanisms that trigger the apoptosis of infected cells followed by phagocytic removal of the apoptotic cells [77]. Phagocyte-mediated clearance of intact apoptotic cells is essential to protect the surrounding tissue against the uncontrolled leakage of cellular contents [78]. Factor H plays an important role in this process by preventing complement activation, membrane attack and cell lysis [71, 79], and its downregulation in FMDV carriers could lead to an uncontrolled release of infectious viral progeny from apoptotic cells.

The two primary mechanisms for the induction of apoptosis in infected cells are T cell-mediated cytotoxicity and death receptor signaling [24]. Our data suggest that both mechanisms are impaired in persistently FMDV-infected tissues from carrier animals. The expression of death receptors, their ligands and other proapoptotic genes in carriers was significantly lower than in non-carriers. At the same time, the expression of antiapoptotic genes was significantly increased and there was evidence for a suppression of the cell-mediated immunity by inducible T_reg_ cells.

Our data and previous serological findings [7, 27, 80, 81] indicate that FMDV carriers have a strong local antibody response. Neutralizing antibodies can reduce the spread of virus between cells, but they cannot eliminate the FMDV-infected cells themselves. Since FMDV is a non-enveloped virus, no viral antigens are exposed on the cell surface that could act as targets for antibody-dependent cell-mediated cytotoxicity.

The strong humoral response in carriers could result from an immune imbalance that favors a T_H_2 response over T_H_1. A possible origin for this T_H_2 bias in persistently FMDV-infected carriers is the overproduction of prostaglandin E_2_ (PGE_2_) and leukotrienes. PGE_2_ inhibits the production of T_H_1 cytokines but not of T_H_2 cytokines, selectively suppressing effector functions of T_H_1 immunity while at the same time promoting T_H_2 responses [82]. An inflammatory process that leads to a local increase of PGE_2_ skews the immune response toward increasingly dominant production of T_H_2-associated cytokines in a positive feedback loop [83], thereby favoring a humoral over a cell-mediated immune response.

Most importantly, PGE_2_ promotes the induction of type 1 regulatory (Tr1) T_reg_ cells by tolerogenic dendritic cells expressing the immunomodulatory cytokines transforming growth factor (TGF) β and interleukin (IL) 10 [82, 84]. IL-10 and TGFβ were overexpressed in NP tissues from carriers compared to non-carriers, and an increase of IL-10 during acute FMDV infection has been previously suggested as a systemic marker of impending FMDV persistence in cattle [85].

Tr1 cells represent a unique subset of antigen-specific regulatory T cells that is distinct from thymus-derived natural T_reg_ cells. Tr1 cells are induced by priming of naïve T lymphocytes with their antigen in the presence of IL-10 [86]. Upon activation, Tr1 cells suppress both naïve and memory T-cell responses through multiple suppressor mechanisms, such as IL-10 and TGF-β as secreted cytokines, and various surface molecules, such as CTLA-4 and PD-1 [87]. IL-10 in particular is critical for the maintenance of persistent viral infections [88], and many viral pathogens specifically exploit the IL-10 pathway to help evade host immunity [89]. The PD-1 pathway also has an important role in limiting the effectiveness of antigen-specific T cells during many persistent infections [90], and it has recently been demonstrated that PGE_2_ acts synergistically with PD-1 in this suppression [91].

It is an intriguing possibility that FMDV-specific Tr1 cells are involved in the observed failure of the cellular immune response in persistently infected NP tissues of carriers. This is supported by several lines of evidence: (1) the expression of the Tr1 effector cytokines IL-10 and TGFβ [51, 92], (2) the activation of the CD200/CD200R and PGE_2_ pathways, which promote Tr1 induction [74, 82], (3) the expression of cytokines and chemokines that induce and attract Tr1 cells, such as IL-10, IL-16, IL-21, IL-35 and CCL28 [55, 56, 58, 93], and (4) the expression of Tr1 surface markers LAG-3, CTLA-4, PD-1, ICOS, GITR, CD137, and CD18 [51, 92].

The immunosuppressive environment created by T_reg_ cells can promote the functional exhaustion of cytotoxic T-cells through chronic exposure to their antigen in combination with inhibitory signals and a lack of CD4 T-cell help [49, 50]. Several inhibitory receptors that are upregulated in terminally exhausted T-cells [28, 94] were significantly overexpressed in persistently infected carriers. However, considering the concurrent overexpression of many T-cell activation markers, it cannot be concluded based on the microarray data alone whether exhausted T-cells are present in persistently FMDV-infected NP epithelium.

Relatively few genes were overexpressed in non-carriers. Fibrinogen-like protein 2 (FGL2) was significantly overexpressed in non-carriers, compared to both naïve animals as well as compared to persistently FMDV-infected carriers. Secretory FGL2 is an immunosuppressive effector molecule of T_reg_ cells, whereas membrane-associated FGL2 has prothrombinase activity [95]. Since the pattern of FGL2 expression in our dataset was diametrically opposed to the expression of many other T_reg_ genes (Fig 5), it likely represents the membrane-bound protein which is found on endothelial cells and activated macrophages.

Another immunomodulating gene, guanylate binding protein 6 (GBP6) was similarly overexpressed in non-carriers. After FGL2, GBP6 was the most strongly upregulated gene in non-carriers compared to naïve controls. GBPs are interferon-induced p65 GTPases that promote oxidative killing and deliver antimicrobial peptides to autophagolysosomes [96], thus, it is conceivable that GBP6 promotes FMDV clearance from NP epithelia.

During the early phase of FMDV infection, T_H_2 activation and a strong antibody response are critical for removing the virus from the circulation and limiting the course of acute disease [3]. However, FMDV carrier animals ultimately fail to eliminate persistent virus from the nasopharyngeal epithelium even though they have high levels of systemic and local neutralizing antibodies [11]. Thus, the same immunological decision [97] that allows the host to quickly clear a systemic infection ultimately promotes subclinical virus persistence in the nasopharynx by favoring a humoral over a cell-mediated immune response.

## Conclusions and Outlook

The phenomenon of persistent FMDV infection of bovine NP epithelium has been described by many investigators [10, 15–17], but the mechanisms underlying this immunological failure remain poorly understood [4, 11, 98]. Much of FMDV vaccinology and policy is based on the existence of asymptomatic infection. Thus, a better understanding of the mechanism of FMDV persistence may guide the development of vaccines and biotherapeutics that prevent or terminate the FMDV carrier state in cattle, a critical step towards the implementation of “vaccinate-to-live” control policies.

Based upon the data presented herein, we propose a working hypothesis for FMDV persistence in the bovine NP, in which a combination of apoptosis inhibition, prostaglandin-E_2_-mediated T_H_2 polarization as well as Tr1- and iTr35-mediated peripheral tolerance and possibly T-cell exhaustion results in a highly localized failure of FMDV-specific cell-mediated immunity. Since there was no significant difference between the transcriptomes of vaccinated versus non-vaccinated carriers, these data suggest that at 21 dpi and beyond, the effects of FMDV carrier status are more profound than the effects of vaccination.

Testing these hypotheses will require a thorough phenotypical and functional analysis of bovine FMDV-specific T cells [99–101], as well as deeper and broader transcriptome characterization informed by a better understanding of the genomics of host and virus. While requisite tools for some of these studies in cattle are still under development [102, 103], other studies are already underway in our laboratory.

## Materials and Methods

### Animals and samples

All cattle were Holstein breed, between 6 and 8 months of age and weighing approximately 200 kg and were obtained from an experimental livestock provider (Thomas D. Morris Inc., Reisterstown, MD, USA) accredited by the Association for Assessment and Accreditation of Laboratory Animal Care International and licensed by the U.S. Department of Agriculture (USDA). All cattle were bred and raised specifically for research.

Naïve steers, vaccinated steers and non-vaccinated steers were housed in separate isolation rooms and were allowed approximately two weeks of acclimation before experiments began. All animal procedures were performed at the Plum Island Animal Disease Center under experimental protocols approved by the Plum Island Institutional Animal Care and Use Committee (protocol numbers 209-12-R, 209-15-R). Animals were fed alfalfa cubes twice daily and had free access to drinking water. The health status of all animals was assessed daily throughout the study period. Based on daily clinical assessments, analgesics and anti-inflammatory drugs (flunixin meglumine, 1.1–2.2 mg/kg; butorphanol tartrate, 0.1 mg/kg) were administered if needed to mitigate pain associated with severe foot-and-mouth disease.

Eighteen out of 20 cattle were challenged by needle-free intranasopharyngeal (INP) deposition [10, 13] in four separate experiments with 10^5^ infectious doses of FMDV strain A_24_ Cruzeiro (titrated in bovine tongue, 50% infectious doses, BTID_50_) [35, 104]. Two weeks before infection, 8 of the 18 challenged animals were immunized using a recently licensed recombinant FMD serotype A vaccine (USDA product code 1FM.1R0; manufactured by Antelope Valley Bios, Lincoln, NE, USA). This vaccine contains the P1-2A and 3C coding regions of FMDV A_24_ Cruzeiro in a replication-deficient human adenovirus serotype 5 vector [105–107]. Half of the vaccinated animals were intramuscularly injected with the licensed product release dose and half with a tenfold higher dose (Table 1). Both doses were delivered in a total volume of 2 mL containing commercially available adjuvant (#7010101, VaxLiant, Lincoln, NE, USA). Studies in vaccinated and non-vaccinated cohorts were performed in parallel, with the same preparation of virus inoculum used for both categories of animals. Two additional non-vaccinated non-challenged animals of similar age from the same herd were housed in a separate isolation room and used as negative controls.

Oropharyngeal fluid (OPF) samples from FMDV-infected animals were collected twice weekly by use of a probang cup [108], starting at 14 dpi in non-vaccinated animals and at 7 dpi in vaccinated animals.

Animals were euthanized by intravenous injection of sodium pentobarbital (86 mg/kg) at either 21 or 35 dpi (Table 1). A standardized necropsy procedure [10, 12] was performed immediately after euthanasia, including collection of samples of nasopharyngeal (NP) epithelium for the microarray analysis. Each tissue sample was split into specimens of approximately 30 mg that were placed in empty screw-cap tubes or screw-cap tubes with 1 mL of pre-dispensed RNA stabilization reagent (RNAlater; Ambion). “Dry” tubes were immediately frozen over liquid nitrogen, while tubes with RNAlater were kept at room temperature for 15 min prior to freezing. Frozen tubes were stored at -70°C until further processing.

OPF and tissue samples were tested for FMDV and FMDV RNA by virus isolation (VI) and real-time RT-PCR as previously described [10, 17, 109].

### RNA extraction

All tissue samples were analyzed by FMDV real-time RT-PCR and VI. Based on the results, either one of four NP tissues (rostral dorsal NP, caudal dorsal NP, rostral dorsal soft palate, or caudal dorsal soft palate) from each animal was selected for the microarray analysis. For non-carriers with consistently real-time RT-PCR- and VI-negative OPF, residual viral RNA in tissue samples in the absence of infectious virus was considered acceptable [109], whereas for carrier animals, only tissues that were positive in the FMDV real-time RT-PCR and VI were used for the microarray.

The selected RNAlater-preserved tissues were thawed on ice, and one piece from each tube was removed at random. Muscle and connective tissue were trimmed off, the remaining epithelial tissue was cut into smaller pieces, and the pieces were suspended in 600 μL RLT buffer (Qiagen) in a 12-mm diameter polypropylene snap-cap tube. The tubes were kept on ice, and the tissue pieces were mechanically sheared in a hand-held rotor-stator homogenizer (Thermo Scientific). Total RNA was extracted from the homogenate with QIAshredder spin columns and the RNeasy Mini Kit (both Qiagen) as directed by the manufacturer. The total RNA concentration was determined with a NanoDrop 1000 spectrophotometer (Thermo Scientific), and RNA integrity was confirmed with RNA 6000 Nano chips on a 2100 Bioanalyzer (Agilent).

### RNA amplification and labeling

RNA was amplified and labeled using the Two-Color Low Input Quick Amp labeling kit (5190–2306; Agilent) following the manufacturer’s recommendations. Briefly, the total RNA template was mixed with RNA spike-in controls (5188–5279; Agilent) and reverse transcribed with AffinityScript RT using an oligo-dT/T7-promoter primer. Cyanine (Cy) 3- and Cy5-labeled antisense complementary RNA (cRNA) was transcribed from the 2^nd^ cDNA strand and purified with a modified RNeasy Mini Kit spin protocol. RNA concentration and specific dye activity was determined with a NanoDrop 1000 spectrophotometer (Thermo Scientific).

### Microarray design and production

The bovine whole genome expression microarray was based on a previously described design [25], with subsequent enhancements. In the current version, it contains 45220 features, 43710 of which are 60-mer sense DNA probes based on non-redundant bovine mRNAs and expressed sequence tags (ESTs) from the NIH genetic sequence database (http://www.ncbi.nlm.nih.gov/genbank/). The probe locations are biased to the 3' end of the sequences in order to produce high signal intensities with the poly-T-primed labeling chemistry. Fifty-eight probes for the FMDV genome were also included in the array to detect viral RNA in samples; all these probes correspond to FMDV polymerase gene (3D) sequences from the NIH database. The remaining features are used for array positioning, background estimation and spike-in controls. Glass slides with four 44K high-density arrays to a slide were produced by a commercial supplier (SurePrint HD, G2514F; Agilent).

The FMDV probe sequences were aligned to the A_24_ Cruzeiro reference sequence (GenBank accession AY593768.1) using the NCBI basic local alignment search tool (http://blast.ncbi.nlm.nih.gov/Blast.cgi). The association between the probe/virus sequence similarities and binding locations from the alignment and the observed signal intensities were examined with Pearson's product-moment correlation coefficient.

### Hybridization and scanning

All total RNA samples used for labeling had an RNA integrity number above 8. Each hybridization reaction contained 2 × 825 ng of fragmented labeled cRNA with a specific activity of at least 12 fmol/ng for each dye.

Cy3- and Cy5-labeled cRNA from two tissue samples was hybridized to paired arrays in a dye-swap arrangement, for a total of four tissue samples per slide. The slide assemblies were incubated for 18 hours at 65°C in a rotating oven set to 10 revolutions per minute. After the hybridization, array slides were washed following the manufacturer’s recommendations, coated with Cy5-stabilization and drying solution (5185–5979; Agilent) and scanned immediately with a GenePix 4000B scanner (Molecular Devices).

Each array image underwent thorough visual inspection to find and eliminate artifacts. Array features were then extracted with GenePix Pro 7.2 using standard settings.

### Background correction and normalization

The median foreground and neighborhood background intensities for all arrays were extracted from the GenePix results files using the *limma* software package [110] in the R/Bioconductor statistical environment [111].

The feature background was corrected using the “normexp” method, which fits a convolution of normal and exponential distributions to the foreground intensities with the background intensities as a covariate. The expected signal given the observed foreground was used as the corrected intensity. This results in a smooth monotonic transformation of the background-subtracted intensities with positive corrected values [112, 113]. A small offset (+10) was applied to the intensities before log-transforming to shrink log-ratios towards zero at the lower intensities and avoid fanning.

Log-ratios within each array were normalized for dye bias to remove systematic trends that arise from the microarray technology rather than from differences between the probes or the RNA hybridized to the arrays [114]. For each array, the log-ratios were adjusted using robustly fitted 5-parameter splines with high breakdown point regression and empirical Bayes shrinkage [110]. Quantile normalization was then used to achieve consistency between arrays, i.e. ensure that the average intensities have the same empirical distribution across arrays leaving the log-ratios unchanged [115].

After background correction and normalization, within-array plots of log-intensity ratios versus log-intensity averages with highlighted control spots were examined for quality control. Pre-processing parameters were adjusted iteratively until all arrays passed visual inspection.

### Statistical analysis

Samples were assigned to one of five groups: non-vaccinated FMDV carriers (n = 7), vaccinated carriers (n = 4), non-vaccinated non-carriers (n = 3), vaccinated non-carriers (n = 4), and naïve controls (n = 2) (Table 1). A separate-channel approach using a common correlation model [116] was used to analyze the two-color array data. The probes were not pre-filtered. For each of the 43768 bovine transcriptome and FMDV genome probes, the (technical) intra-spot correlation between the channels was estimated across all arrays and used to fit a linear model to the data in terms of the individual log-intensities. Once the linear model was fitted, inference proceeded in the same way as for log-ratios in a fully connected experimental design [116].

### Group-wise comparisons

Using the linear model, contrast matrices were set up for three comparisons: one within the FMDV-infected animals alone (non-carriers vs. carriers) and two between FMDV-infected animals and naïve controls (i.e., all non-carriers vs. naïve controls and all carriers vs. naïve controls). For each contrast and probe, log_2_ fold changes of signal intensity, empirical Bayes moderated t-statistics and p-values were calculated as previously described [117]. Unless stated otherwise, all fold change analyses are based on the log_2_ values (log_2_ fold change, log_2_FC).

To account for multiple testing within a contrast, p-values were adjusted using the Benjamini and Hochberg [118] method to control the false-discovery rate (FDR). Values adjusted with this method are bounds on the FDR rather than rejection probabilities in the usual sense of a p-value, and they are referred to as q-values. A q-value of less than 0.1 was considered significant; accordingly, the expected proportion of false discoveries is controlled to be less than 10% [119].

For each probe, three fold-change values are reported: the difference in signal intensity (as a proxy for the expression of the corresponding gene) between persistently FMDV-infected carriers and naïve controls, between non-carriers and naïve controls, and between non-carriers and carriers.

Most of the subsequent analyses are based on the relative difference in signal intensity (i.e., the predicted log-ratio) between non-carriers and carriers. In this comparison, probes that have higher signal intensity in non-carriers have positive fold-change values; negative values reflect probes that had higher signal intensities in carriers. For each probe, the association of the fold changes in non-vaccinated and vaccinated animals was estimated using Pearson’s product-moment correlation coefficient. In addition, the biological correlation between the differential gene expression in non-vaccinated and vaccinated animals was calculated by separating the biological from the technical components of the correlation [120].

Signal intensities in non-carriers and carriers were each compared to the naïve control animals to establish absolute differences in expression (over/under presumed normal) for each corresponding gene. In this comparison, the predicted fold changes are reported relative to the naïve controls, i.e. a positive fold change indicates stronger expression than in the controls, and vice versa.

The figures and supplemental tables only show probes with at least a 50% difference in signal intensity (absolute log_2_FC >0.58) between non-carriers and carriers and an associated q-value of <0.1; probes are ranked by decreasing magnitude of difference between non-carriers and carriers.

### A priori selected gene sets

In addition to the ranked lists of probes, sets of probes (genes) related to apoptosis, T-cell exhaustion and related inhibitory receptors, regulatory T cells, eicosanoid synthesis, as well as the alternative complement pathway were selected based on a literature search. If more than one probe matched a given gene, only the probe with the lowest p-value in the non-carrier/carrier comparison was included in the analysis. The probes are grouped by functional considerations and listed in arbitrary order. Their average signal intensities across the whole data set, as well as the log_2_FC, p- and q-values for the comparison between non-carriers and carriers are shown in the tables regardless of whether they meet the aforementioned log_2_FC or q-value thresholds.

### Unsupervised clustering and gene expression heat map

The background-corrected and normalized intensities of the *a priori* selected genes (probes) were also used for an unsupervised two-dimensional cluster analysis. Probe- and animal-wise distance matrices were calculated based on Spearman's rank correlation coefficient (1 – r) and Manhattan (rectilinear) distance, respectively [121]. Based on the distance matrices, probes and animals were grouped by agglomerative algorithms using either complete linkage or Ward's method, respectively. The sequence of cluster assignments was used to construct unrooted dendrograms illustrating the established relationships between animals or probes, and the dendrograms were used to reorder the rows and columns of a heat map showing the individual probe intensities.

For the heat map, the log_2_-transformed intensities were centered and scaled to a probe-wise mean of 0 and a standard deviation of 1. Cell colors in the heat map correspond to the centered and scaled intensity values (probe-wise z-scores). Cells with negative scores (intensities lower than the overall mean for any given probe) are shaded red and cells with positive scores (higher intensities) are shaded blue. The colored sidebar above the heat map indicates the persistence status of each animal (carriers are shown in red, non-carriers in blue and naïve controls in black).

### Gene group functional profiling

Statistical enrichment analysis using two sources of functional evidence–Gene Ontology (GO; http://geneontology.org/) terms and biological pathways from the Kyoto Encyclopedia of Genes and Genomes (http://www.genome.jp/kegg/)–was performed with the g:GOSt web tool in the g:Profiler suite (http://biit.cs.ut.ee/gprofiler/) [122]. Enriched GO terms were further visualized with GOrilla (http://cbl-gorilla.cs.technion.ac.il/) [123].

The genes in each set (genes that were higher expressed in carriers than in non-carriers, and genes that were higher expressed in non-carriers than in carriers) were ordered by their absolute log_2_FC for incremental enrichment analysis. Only probes with an absolute log_2_FC >0.58 and a q-value <0.1 were included in the analysis. Using a ranked gene list identifies specific functional terms that are associated with the most dramatic changes in gene expression, as well as broader terms that characterize the gene set as a whole.

Hierarchical best-per-parent filtering in g:Profiler was used to obtain a compact representation of gene list enrichment results from the Gene Ontology (GO). Statistically enriched GO terms that shared common parent terms were grouped, and only the sibling term with the strongest p-value is included in the output. The output is sorted by p-value within each evidence source.

## Supporting Information

S1 Fig Correlation between log_2_FC values (non-carriers/carriers) in vaccinated and non-vaccinated animals. (PDF)

S2 Fig UCSC Genome Browser output for bosTau8, chromosome 23, position 27,535,000–27,544,000. (PDF)

S1 Table Differential gene expression between non-carriers and carriers (NC/CR). The probes are sorted by the values in the highlighted log_2_FC column. Cells with log_2_FC values are shaded based on a 3-color gradient between lowest/most negative (red) and highest/most positive (blue), centered on zero (white). Q-values <0.1 are shown in bold type. (avg. signal: average signal intensity across all arrays, log_2_FC: log_2_ fold change, q: adjusted p-value associated with each log_2_FC value).
(XLSX)

S2 Table Differential gene expression between non-carriers and naïve controls (NC/CO). The probes are sorted by the values in the highlighted log_2_FC column. Cells with log_2_FC values are shaded based on a 3-color gradient between lowest/most negative (red) and highest/most positive (blue), centered on zero (white). Q-values <0.1 are shown in bold type. (avg. signal: average signal intensity across all arrays, log_2_FC: log_2_ fold change, q: adjusted p-value associated with each log_2_FC value).
(XLSX)

S3 Table Differential gene expression between carriers and naïve controls (CR/CO). The probes are sorted by the values in the highlighted log_2_FC column. Cells with log_2_FC values are shaded based on a 3-color gradient between lowest/most negative (red) and highest/most positive (blue), centered on zero (white). Q-values <0.1 are shown in bold type. (avg. signal: average signal intensity across all arrays, log_2_FC: log_2_ fold change, q: adjusted p-value associated with each log_2_FC value).
(XLSX)
