# Supplementary figures and images for: Transcriptomic Analysis of Persistent Infection with Foot-and-Mouth Disease Virus in Cattle Suggests Impairment of Apoptosis and Cell-Mediated Immunity in the Nasopharynx
Source: PLoS One. 2016 Sep 19;11(9):e0162750. doi: 10.1371/journal.pone.0162750 (PMC5028045; doi:10.1371/journal.pone.0162750)

Fig. S2: UCSC Genome Browser output for bosTau8, chromosome 23, position 27,535,000-27,544,000.

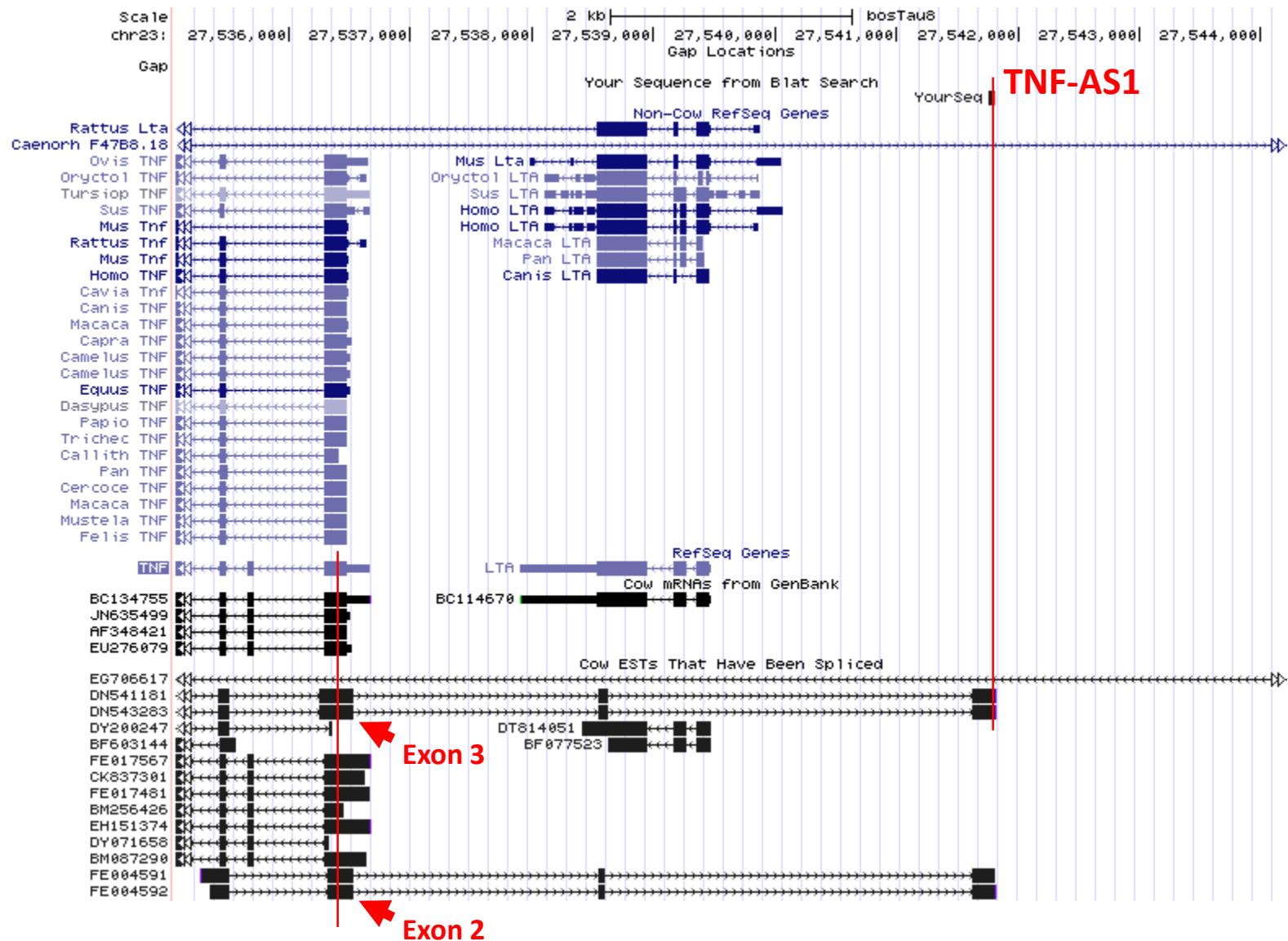

Supplement: S2 Fig — (PDF) [file pone.0162750.s002.pdf]
